# Congenital Syphilis Presenting with Brain Abnormalities at Neuroscan: A Case Report and a Brief Literature Review

**DOI:** 10.3390/microorganisms10081497

**Published:** 2022-07-25

**Authors:** Gabriele Tonni, Gianpaolo Grisolia, Marlene Pisello, Paolo Zampriolo, Valeria Fasolato, Paola Sindico, Edward Araújo Junior, Maria Paola Bonasoni

**Affiliations:** 1 Department of Obstetrics and Neonatology and Researcher, Istituto di Ricovero e Cura a Carattere Scientifico (IRCCS), AUSL Reggio Emilia, 42122 Reggio Emilia, Italy; 2 Department of Obstetrics and Gynecology, Carlo Poma Hospital, ASST Mantova, 46100 Mantova, Italy; grisoliagp@gmail.com (G.G.); marlene.pisello@asst-mantova.it (M.P.); paolo.zampriolo@asst-mantova.it (P.Z.); 3 Neonatal Intensive Care Unit (NICU), Carlo Poma Hospital, ASST Mantova, 46100 Mantova, Italy; valeria.fasolato@asst-mantova.it (V.F.); paola.sindico@asst-mantova.it (P.S.); 4 Department of Obstetrics, Universitade Federal de Sao Paulo, Sao Paulo 04021-001, Brazil; araujojred@terra.com.br; 5 Pathology Unit, Istituto di Ricovero e Cura a Carattere Scientifico (IRCCS), AUSL Reggio Emilia, 42122 Reggio Emilia, Italy; paolabonasoni@yahoo.it

**Keywords:** congenital syphilis, primary syphilis, ultrasound, neurosonography, high-risk pregnancy

## Abstract

A case of vertical transmission in a 35-year-old pregnant woman, gravida 4, para 2 with an unknown medical history of carrying primary syphilis is described. A routine 3rd trimester scan was performed at 30 + 5 weeks of pregnancy, which revealed fetal growth restriction (FGR) associated with absent fetal movement, a pathologic neuroscan characterized by cortical calcifications and ominous Doppler waveform analysis of the umbilical artery and ductus venosus. Computerized electronic fetal monitoring (EFM) showed a Class III tracing, according to the American College of Obstetricians and Gynecologists (ACOG) guidelines. An emergency C-section was performed and a female newborn weighing 1470 g was delivered. The Apgar scores were 5 and 8 at the first and fifth min, respectively. Besides the prompted obstetrical and neonatal interventions, the neonate died after 7 days. A histologic examination of the placenta revealed a chorioamnionitis at stage 1/2 and grade 2/3. The parenchyma showed diffuse delayed villous maturation, focal infarcts, and intraparenchymal hemorrhages. The decidua presented with chronic deciduitis with plasma cells. The parents declined the autopsy. Congenital syphilis is an emerging worldwide phenomenon and the multidisciplinary management of the mother and the fetus should be mandatory.

## 1. Introduction

Syphilis is a sexually transmitted disease (STD) caused by the bacterium *Treponema pallidum*. *T. pallidum* presents an outer membrane devoid of lipopolysaccharides, but abundant lipoproteins are found underneath the surface. The scarcity of surface-exposed pathogen-associated molecular patterns (PAMPs) allows the spirochaete to elude the hosts immune system, favoring local replication and dissemination. *T. pallidum* low surface antigenicity prevents antibody recognition, which allows for prolonged persistence. Bacterial latency can persist for years, maintaining nidus of inflammation in skin, bones, the thoracic aorta, the posterior uveal tract, and the central nervous system paving the path for later recrudescence. In the *T. pallidum* genomic sequence, 12-membered repeat (Tpr) proteins have been identified as part of the outer sheath. Among these, TprK might play a key role in eluding the host immune response due to antigenic variation in seven regions [1].

Syphilis has a global incidence of approximately 6 million cases each year and most infection occurs in low and middle-income countries. In high-income countries, syphilis persists in poor people, marginalized subjects who do not have access to healthcare, and in racial or ethnic groups. Although infrequent, in high-income countries syphilis spreads within tight social or sexual networks, especially between men who have sex with other men (MSM), or subjects with multiple partners, who can easily switch to the heterosexual population, without health control [2,3]. The global estimate for syphilis prevalence in MSM has been calculated at 7.5%, a significantly increased percentage between 2000 and 2020 [4]. On the other hand, heterosexual transmission is the main cause of infection in childbearing women, favoring the recent growing number of congenital syphilis cases [5]. In the United States, the prevalence and incidence have dramatically increased between 2008 and 2018 from 164% and 175%, respectively. In 2018, there were 156,000 and 146,000 prevalent and incident infections in individuals aged 14–49, respectively. Men were estimated around 70% of the prevalent syphilis and 80% of the incident [6]. In the European Union/European Economic Area (EU/EEA) in 2018, 30,000 syphilis cases were reported to the European Centre for Disease Control, with a rate of 6.1/100,000, and the men’s prevalence was 8 times more when compared to women [7]. Along with these figures, the cases of congenital syphilis have been rising accordingly. Between 2012 and 2016, the global maternal syphilis rate was 0.69% and 0.70%, respectively [8]. In the United States in 2018, the incidence was 33 cases per 100,000 live births [5]. In 2018, 23 EU/EEA countries reported 60 congenital syphilis newborns, indicating a rate of 1.6/100,000 live births. Compared to 2015 and 2017, in which the rate was 1.1 and 1.2/100,000 live births, the cases had been slightly exacerbating [9].

Vertical transmission from the mother to the fetus can occur throughout pregnancy as a consequence of placental infection. More rarely, primary syphilis may be secondary to the passage of the bacterium to the fetus from injuries of the vaginal mucosa or through breastfeeding in cases of breast lesions [10]. Congenital syphilis due to mother-to-child transmission can promote multiple fetal structural abnormalities considering the combination of bacterial high neurotropism and placental pathology. The latter may induce fetal cerebral lesions to the developing brain and placental dysfunction may cause fetal growth restriction (FGR) and high-risk poor perinatal outcome. Affected newborns can develop long-term sequelae, such as deafness and neurological impairment [11]. The gold-standard of care for either treatment and prevention of transmission relies on Penicillin G and a general antibiotic regimen should be started once the clinical diagnosis of syphilis has been established. However, a high percentage of infants, almost 50% and 35%, will result affected from mothers with early, untreated, or latent stage disease [12,13]. 

Mothers, without an appropriate antibiotic regimen started promptly, are at a 12-fold increased risk of adverse pregnancy outcomes when compared with controls [14], which is in keeping with a previous observation by Lubiganon et al. [15]. A systematic review with meta-analysis documented that untreated pregnancies had an increased statistically significant risk of adverse outcomes when compared to controls (76.8% vs. 13.7%, *p* = 0.000) [16]. 

Nowadays, syphilis represents a worldwide warning for healthcare systems as the prevalence of the disease is increasing with 1.9 million affected mothers worldwide [17]. With this *scenario*, mother-to-child transmission is responsible for nearly 305,000 perinatal deaths every year [18] and currently primary syphilis is one of the common life-threatening perinatal infectious disease [19]. Programs based on responsible and safety intimate behavior, antenatal serologic screening tests, monthly antenatal clinic examination, as well as timely and available treatment should be mandatory in controlling and reducing infection [20].

## 2. The Case

**Figure 1:**
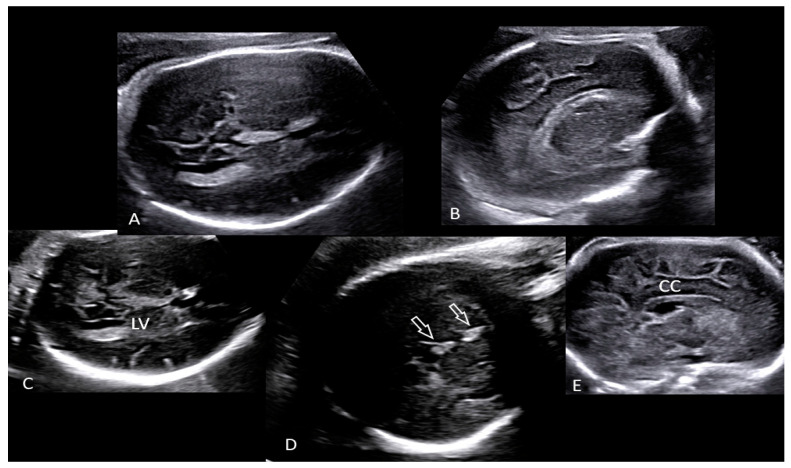
Transabdominal neuroscan (**A**–**E**) performed in the delivery room detected hyperechogenicity (**D**, open arrows) involving both choroid plexuses of the lateral ventricles (LV) as well as the *falx cerebri*. Brain contour was smooth without recognizable gyri and sulci (**A**,**B**,**E**). (Legend: CC: corpus callosum; LV: lateral ventricle).

**Figure 2:**
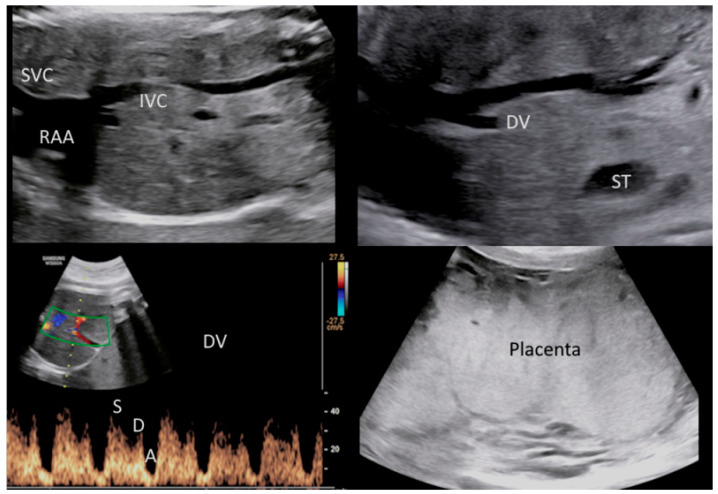
Doppler ultrasound revealed abnormal venous flow of the ductus venosus (DV) with a PI: 1.25. These findings were suspicious for metabolic, infective and/or hypoxic-ischemic events. (Legend: A: atrial contraction; D: diastole; DV: ductus venosus; IVC: inferior vena cava; PI: pulsatility index; RAA: right atrium appendage; ST: stomach; SVC: superior vena cava; S: systole). Ultrasound also documented a placentomegaly.

**Figure 3:**
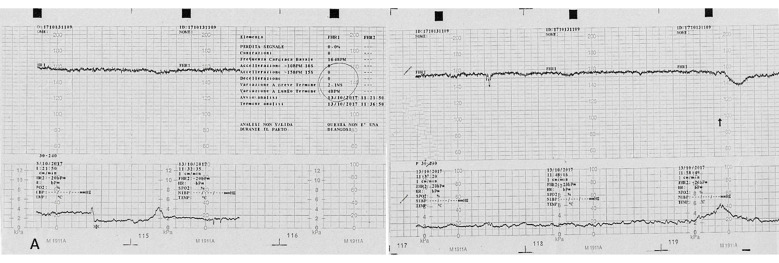
Computerized electronic fetal monitoring (EFM) during antepartum: a baseline fetal heart rate of 164 bpm is visible associated to an absent long-term variability (<5 bpm) and with shallow late deceleration (Class III American College of Obstetricians and Gynecologists classification).

**Figure 4:**
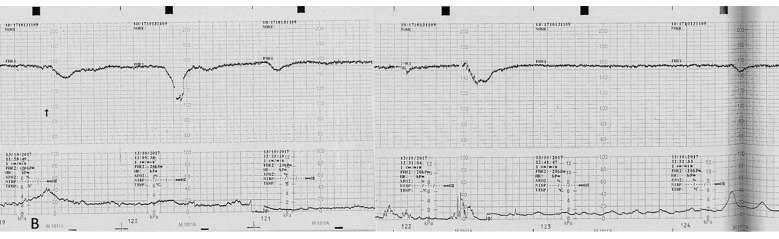
Computerized electronic fetal monitoring (EFM) during antepartum showing absent long-term variability (<5 bpm) associated with recurrent shallow late deceleration (Class III American College of Obstetricians and Gynecologists classification).

A pregnant 35-year-old woman, G4P2, was referred for a routine third trimester scan. The current pregnancy was spontaneous and was complicated by gestational diabetes mellitus (GDM) under diet control. The mother attended regular antenatal clinic and laboratory exams. Serological tests were made during the first trimester, according to the Italian Ministerial Decree (DPCM 12 January 2017) [21], including non-treponemal test (NTTs) and treponemal tests (TTs) for the detection of syphilis, which were performed and resulted negative. Fetal karyotype was performed for advanced maternal age by chorionic villus sampling (CVS) and was found normal. The third trimester scan carried out at 30 + 5 weeks of gestation revealed a fetus in breech presentation with ultrasound signs of fetal growth restriction (FGR) (biometry parameters were the 5th percentiles for gestational age, according to Società Italiana di Ecografia Ostetrica e Ginecologica-SIEOG 1986 charts) [22] and absent fetal movements. A transabdominal neuroscan was able to detect hyperechogenicity involving both choroid plexuses of the lateral ventricles as well as the *falx cerebri*. Her brain contour was smooth without recognizable gyri and sulci (Figure 1). The amount of amniotic fluid assessed using amniotic fluid index (AFI) was within normal ranges. A Doppler ultrasound investigation of the umbilical artery showed a pulsatility (PI) and resistance indices (RI) of 1.44 and 0.78, respectively. The end-diastolic flow (EDF) was absent. The fetal cerebral Doppler were in normal ranges: PI: 1.62, RI: 0.82, Vmax: 42.9 cm/s, and positive EDF of the middle cerebral artery (MCA). However, the Doppler ultrasound revealed abnormal venous flow at the level of the ductus venosus (DV) documented by a PI: 1.25 and by the presence of a A-wave (atrial contraction) (Figure 2). These Doppler findings were suspicious for metabolic, infective, and/or hypoxic events. The mother was admitted to the hospital and immediately sent to the delivery room. On admission, she had regular vital signs and an unremarkable physical examination. Clinical tests showed the following: hemoglobin (Hb): 11 g/L, white blood count (WBC): 7370/μL, platelets (PTLs): 266,000/μL, serum albumin: 29 g/L, and C-reactive protein (CRP): 70.9 mg/L. The electronic fetal monitoring (EFM) documented a Class III American College of Obstetricians and Gynecologists (ACOG) tracing [23]: baseline fetal heart rate (FHR) of 164 bpm, no accelerations with absent variability (2.1 ms at the computerized evaluation) associated with shallow late decelerations (Figure 3 and Figure 4). According to these parameters, an emergency C-section was performed following intravenous administration of magnesium sulfate 4 g for fetal neuroprotection. 

A 1470 g female newborn was delivered with an Apgar score of 5 and 8 at the first and fifth minutes, respectively. An acid-base assessment was determined on the umbilical artery and vein and showed a pH of 7.1 and 7.07, respectively, while base excess (BE) was similar in both cord vessels (−13.8 mEq/L). The neonate was transferred to neonatal intensive care unit (NICU) for assisted ventilation. General antibiotics, antiviral, and antifungal prophylaxis was performed. Stomach and umbilical vein bleedings were recorded and blood samples showed severe blood clot anomalies (INR: 6.15; aPTT: 3.12; C-protein: 28%), thrombocytopenia (PTLs count < 4000/ µL), and hypoglycemia (19 mg/dL) that were treated with fresh frozen plasma, platelet transfusion, C-protein, and glucose infusion. The CRP was 157 mg/L (normal value: <10 mg/L). 

A transfontanellar ultrasound scanning revealed a bilateral intraventricular hemorrhage (grade III IVH) [24] that progressed to grade IV during hospital admission. Clinical signs of hypoxic-ischemic encephalopathy (HIE) with seizures during the first 24 h developed and resolved using phenobarbital, midazolam, and phenytoin administration. However, end-organ injuries ensued with pulmonary hemorrhage, anemia, cholestasis, systemic hypotension, and renal failure. Signs of end-organ failure were promptly treated with intubation, high frequency ventilation, blood, platelet and plasma transfusions, dopamine, albumin, and furosemide administration. A laboratory investigation performed at 24 h from birth confirmed a positive syphilis test, and an intramuscular benzathine benzylpenicillin injection regimen was started. Unfortunately, the clinical conditions worsened and the newborn died at 7 days of life. The mother was treated with benzathine benzylpenicillin G (2,400,000 IU) intramuscularly (IM) and was discharged in good condition on day four. At the time of writing, the woman was followed up at 4 months: physical examinations were unremarkable following completion of the antibiotic regimen and laboratory test for *T. pallidum* showed negative IgM and high-titer IgG levels at TTs test. To the best of our knowledge, the woman has not conceived again yet.

Serologic tests for syphilis showed a profile compatible with a primary, acute infection disease, as the rapid plasma regain (RPR) was positive at a title of 64 (reference value: <1). Furthermore, *T. pallidum* tests (TTs) that detect IgM and IgG antibodies specific to *T. pallidum* reported positive at a title of *T. pallidum* agglutination assay (TPPA) > 5120 (reference value < 80). Infectious diseases including the detection of HIV, Hepatitis B and C, Herpes simplex virus 1-2, Cytomegalovirus, *Listeria monocytogenes,* and Rubella were all negative.

**Figure 5:**
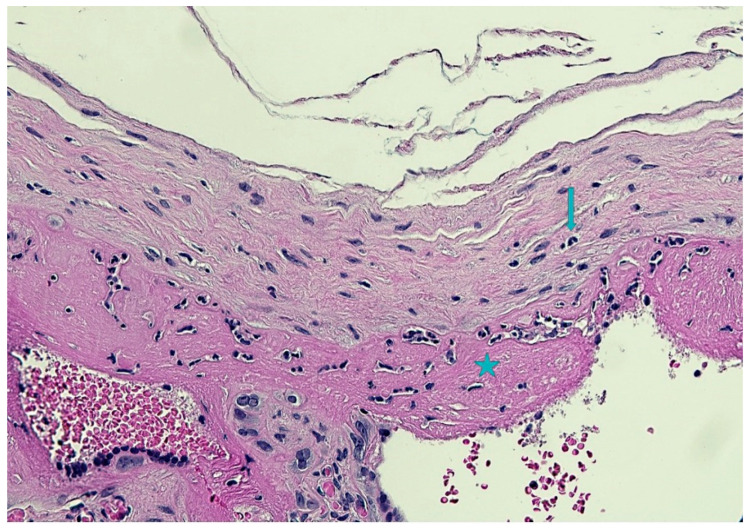
Subchorionitis with focal chorioamnionitis: granulocytes were mainly located in the subchorion (star) with focal extension into the chorion (arrow)(Hematoxylin and Eosin staining, 10 HPF).

The placenta was sent for histologic examination. Unfortunately, the parents declined the autopsy on the newborn. Macroscopically the placenta weighed 678.0 g (97th percentile for the gestational age) [25] and measured 20.0 × 13.0 × 3.0 cm. The umbilical cord measured 28.0 cm in length with three vessels and a central insertion into the chorionic plate. The umbilical coil index (UCI) was 0.25, just under the upper limit of normality (0.3). Recent hemorrhagic suffusion was noted in the Wharton’s jelly. Histological examination showed chorioamnionitis stage 2/3 and grade 1/2 [26]. Granulocytes were mainly located in the subchorion with focal extension into the chorion (Figure 5). 

**Figure 6:**
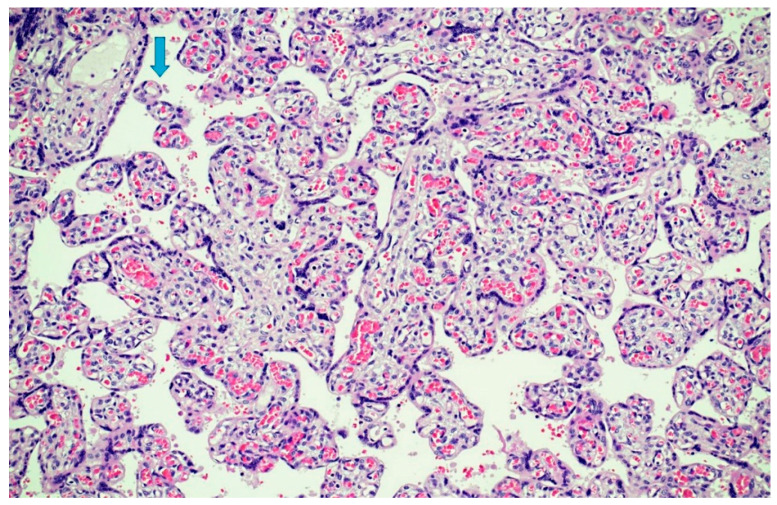
Parenchymal dysmaturity: chorionic villi showed diffuse delayed villous maturation with plump villi and rare terminal villi (arrow)(Hematoxylin and Eosin staining, 4 HPF).

**Figure 7:**
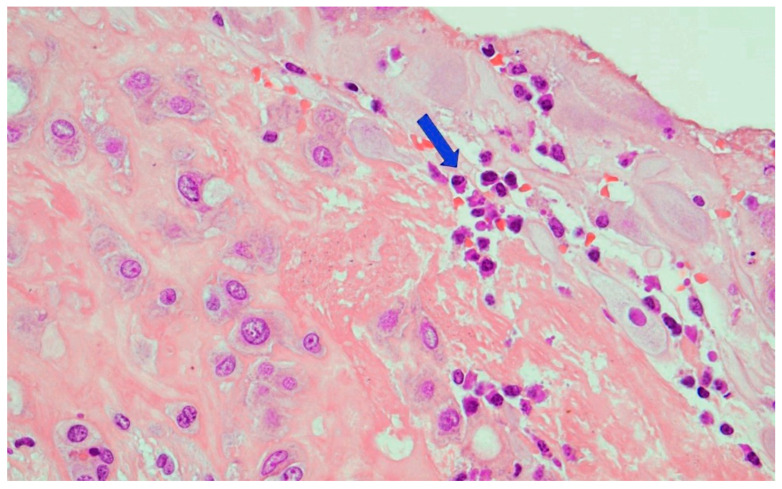
Chronic deciduitis: the decidua showed numerous plasma cells (arrow)(Hematoxylin and Eosin staining, 20 HPF).

Funisitis was absent. Parenchyma presented a diffuse delayed villous maturation (Figure 6), focal infarcts, and intraparenchymal hemorrhages at different stages of organization. The decidua presented chronic deciduitis with plasma cells (Figure 7). Immunohistochemistry for *T. pallidum* resulted as negative. 

## 3. Discussion 

The pathogenetic mechanism underlying the syphilis vertical transmission has been investigated by Hollier et al. in 2001 [27] and later by Rac et al. [28]. It seems that *T. pallidum* affects the fetus *via* a placental and liver colonization with further shedding into the amniotic fluid. At a later stage, blood dysfunction, such as anemia, may ensue, as well as congestive heart failure contributing to ascites with fetal IgM production. A massive *T. pallidum* colonization of both the placenta and fetal liver explains the increased placenta and liver volume. This series of processes are time-dependent and mothers not recognized of carrying the disease and thus left untreated are at a higher risk that their unborn fetuses could potentially develop congenital malformations [27,29]. Prenatal ultrasound is the first-line of diagnostic investigation in pregnancy and can be used to follow up with the fetus at the time of the identification of syphilis infection. A thorough ultrasound examination, and specifically a targeted-neurosonograhy, may demonstrate that typical findings of late mother-to-child transmission, e.g., middle cerebral artery (MCA) Doppler abnormalities and ascites may improve over time whilst increased placental and liver volume require a longer time to recover after antibiotic therapy [28].

Serologic testing relies upon detection of both NTTs and TTs antibodies. NTTs include RPR and the venereal disease research laboratory (VDRL) test, which detects both IgM and IgG antibodies against cardiolipins released from host cell damage during infection. These tests can be qualitative or quantitative with titers that increase with acute disease and decrease following specific therapy regimen. Higher NTT titers are seen in primary and secondary syphilis as compared to latent syphilis. Treponemal tests (TTs) include all assays that detect IgM and IgG antibodies specific to *T. pallidum*. While these tests can confirm previous *T. pallidum* infection, they cannot differentiate individuals who have been treated from those with current disease. Generally, TTs remain reactive for life following the eradication of the infection. Historically, the most commonly used TTs were the TPPA and the fluorescent treponemal antibody absorption (FT-ABS) assay. Recent advances in the detection of *T. pallidum* antibodies have resulted in several TTs that are highly sensitive and specific [30]. 

In order to maximize detection rates, the Centers for Disease Control and Prevention (CDC) recommends screening all pregnant women at the beginning of prenatal care, while mothers at high-risk should be screened twice during the third trimester and again at delivery [31]. When pregnant women are found positive with laboratory testing for syphilis, especially for primary syphilis, a detailed antenatal clinical examination and serologic tests for other sexually transmitted infections are mandatory. Furthermore, a multi-specialist counseling should be planned and undertaken by obstetricians with known expertise in high-risk pregnancy and specialists in infectious diseases.

An extended, thorough ultrasound examination is the primary diagnostic tool in identifying potential signs of intrauterine transmission [27,29,32,33,34]. Although vertical transmission does not determine specific ultrasound clusters of the disease, fetuses with ultrasound signs consistent with congenital infection are more prone to undergo *in utero* and *ex utero* complications [28,35]. 

As stated before, placental and liver colonization by *T. pallidum* jeopardize the fetus and are directly responsible of increased hepatic (80%) and placental volume (27%). Moreover, they secondarily cause organ dysfunctions, such as elevated Doppler ultrasound peak systolic velocity (PSV) of the middle cerebral artery (33%, an indirect sign of fetal anemia), hydramnios (12%), and ascites or fetal hydrops (10%) [27,29,34].

Notwithstanding, a normal ultrasound is not sufficient, per se, to exclude intrauterine infection, as it has been demonstrated that nearly 12% of infants with a negative scan underwent antibiotic treatment for mother-to-child transmission at delivery. 

CDC guidelines claim that the only effective treatment for preventing mother-to-child transmission is penicillin G, according to the maternal stage of infection. Pregnant women allergic to penicillin should be desensitized and treated consequently. In adults, the recommended treatment for primary, secondary, and early syphilis is benzathine benzylpenicillin G 2.4 million units IM in a single dose. In late latent and tertiary syphilis (with normal cerebrospinal fluid examination) the treatment consists of benzathine benzylpenicillin G 7.2 million units in total, provided as 3 doses of 2.4 million units IM each at 1 week intervals.

In pregnancy, women with primary, secondary, or early latent syphilis, an additional dose of benzathine benzylpenicillin G 2.4 million units may be given 1 week after the first dose in order to avoid fetal transmission. The same dose should be administered in case of US signs of congenital syphilis (hydrops, hepatomegaly, placentomegaly), especially during the second trimester of pregnancy, as an additional beneficial effect for the fetus. Strict adhesion to the treatment plan is mandatory and doses must not be missed. The best interval is one week with a benefit of two days. In case of a missed dose, full therapy must be repeated [31]. 

The grade of severity of primary congenital syphilis infection is related to the time of onset, diagnosis, and treatment [28,29,31]. When mothers are diagnosed with primary congenital infection, counseling should be undertaken by the multi-specialists team in order to provide accurate information on prenatal and postnatal management strategy. Ultrasound examination should be performed by expert sonographers and includes an extended examination with targeted neuroscan and Doppler ultrasound [27,29,36,37].

The fetal liver is particularly affected by the *T. pallidum* infection *in utero*, and may develop a series of pathology ending in end-organ injury or even therapeutic paradox after syphilis therapy has been started [38,39,40]. Increased placental volume is another dramatic sign secondary to placental infection [41,42,43] and contributes to an increased utero-placental resistance [44]. 

As a result, histologic examination of the placenta should be considered when facing mothers and newborns with proven primary vertical transmission. Usual histological findings include chorioamnionitis, funisitis, villous dysmaurity/immaturity, acute and/or chronic villitis, chronic deciduitis, villous dysmaturity/immaturity, and Hofbauer cell hyperplasia [40,44]. Regarding fetal autopsy, the most common anomaly reported, other than hepatomegaly and hydrops, was stress-related thymus anomalies, such as starry-sky appearance and cortical shrinking. Brain inflammation with necrosis was rarely observed [44]. In general, brain abnormalities were scarcely detected, even at prenatal US, although a high MCA PSV was highly indicative of fetal anemia [36]. Although untreated syphilis in pregnancy is a recognized risk factor for miscarriage and stillbirth, only one case of microcephaly was described [45,46,47,48].

## 4. Conclusions

As we have seen, an increase of syphilis is an emerging worldwide phenomenon that requires particular attention, as it is associated with potential serious anomalies in cases of primary maternal infections. Laboratory screening, regular antenatal clinical examination, and treatment should be the gold-standard of care for all the professionals involved in prenatal and perinatal management when facing mothers with a positive serologic test. Clinical and laboratory follow up are also recommended in order to establish the specific immunologic assessment following primary and/or secondary syphilis infection.

## Data Availability

The data presented in this study are available on request from the corresponding author.
